# Expression and inhibition of BRD4, EZH2 and TOP2A in neurofibromas and malignant peripheral nerve sheath tumors

**DOI:** 10.1371/journal.pone.0183155

**Published:** 2017-08-15

**Authors:** Azadeh Amirnasr, Rob M. Verdijk, Patricia F. van Kuijk, Walter Taal, Stefan Sleijfer, Erik A. C. Wiemer

**Affiliations:** 1 Department of Medical Oncology, Erasmus MC Cancer Institute, Erasmus University Medical Center, Rotterdam, The Netherlands; 2 Department of Pathology, Erasmus University Medical Center, Rotterdam, The Netherlands; 3 Department of Neuro-Oncology / Neurology, Erasmus University Medical Center, Rotterdam, The Netherlands; Universidad de Navarra, SPAIN

## Abstract

Malignant peripheral nerve sheath tumors (MPNST) are rare, highly aggressive sarcomas that can occur spontaneously or from pre-existing plexiform neurofibromas in neurofibromatosis type1 (NF1) patients. MPNSTs have high local recurrence rates, metastasize easily, are generally resistant to therapeutic intervention and frequently fatal for the patient. Novel targeted therapeutic strategies are urgently needed. Standard treatment for patients presenting with advanced disease is doxorubicin based chemotherapy which inhibits the actions of the enzyme topoisomerase IIα (TOP2A). Recent molecular studies using murine models and cell lines identified the bromodomain containing protein 4 (BRD4) and enhancer of zeste homolog 2 (EZH2) as novel targets for MPNST treatment. We investigated the expression and potential use of BRD4, EZH2 and TOP2A as therapeutic targets in human NF1-derived MPNSTs. The transcript levels of *BRD4*, *EZH2* and *TOP2A* were determined in paired formalin-fixed paraffin-embedded (FFPE) neurofibroma/MPNST samples derived from the same NF1 patient and in a set of plexiform neurofibromas, atypical neurofibromas and MPNST. We further examined the effect on cell viability of genetic or pharmacological inhibition of BRD4, EZH2 and TOP2A in an MPNST cell line panel. Our results indicated that in MPNST samples *BRD4* mRNA levels were not upregulated and that MPNST cell lines were relatively insensitive to the bromodomain inhibitor JQ1. We corroborated that *EZH2* mRNA expression is increased in MPNST but failed to confirm its reported pivotal role in MPNST pathogenesis as EZH2 knockdown by siRNA did not interfere with cellular proliferation and viability. Finally, the relation between TOP2A levels and sensitivity for doxorubicin was examined, confirming reports that *TOP2A* mRNA levels were overexpressed in MPNST and showing that MPNST cell lines exhibited relatively high TOP2A protein levels and sensitivity to doxorubicin. We tentatively conclude that the potential for effective therapeutic intervention in MPNST by targeting BRD4, EZH2 and TOP2A individually, may be limited. Clinical studies are necessary to ultimately prove the relevance of BRD4 and EZH2 inhibition as novel therapeutic strategies for MPNST.

## Introduction

Neurofibromatosis type 1 (NF1) is an autosomal dominant disorder which has a *de novo* incidence of one in 3000 individuals [1–3]. This genetic disorder is caused by defects in the *NF1* gene located on chromosome 17q11.2. The *NF1* gene encodes a tumor suppressor called neurofibromin 1, which through its GTPase-activating protein (GAP) domain negatively regulates Ras signaling keeping cell proliferation in check. Inherited or sporadic mutations of *NF1* and the partial inactivation of neurofibromin, lead to an increased risk of developing various tumors. Almost all NF1 patients develop cutaneous neurofibromas and in many patients plexiform neurofibromas cause additional morbidity. All tumors exhibit biallelic inactivation of the *NF1* gene and consequently activated signaling through the Ras pathway driving cancer formation [1, 4]. Plexiform neurofibromas may transform into malignant peripheral nerve sheath tumors (MPNST), the most common malignancy occuring in NF1 patients, at an incidence of 2% and a lifetime risk of 8–13% [5]. MPNSTs are classified in the group of the soft tissue sarcomas (STS) and comprise approximately 5–10% of all STS. MPNST are a class of highly aggressive and clinically challenging sarcomas. High local recurrence rates, early metastasis and resistance to chemotherapy are common clinical phenotypes in this cancer. When metastasized, patients face a poor prognosis with only a limited number of systemic chemotherapeutic agents available [6, 7]. Of these, doxorubicin is probably the most active one, targeting—through intercalation into the DNA—the activity of the enzym topoisomerase IIα (TOP2A) [8]. Transcriptome data analyses have shown that *TOP2A* was among the most upregulated genes in MPNSTs when compared to benign neurofibromas [9, 10]. However, despite the high expression of TOP2A, advanced MPNST patients do not respond well to doxorubicin given a 2 year overall survival rate of approximately 20%, which is roughly equivalent to the outcome of patients with metastatic STS other than MPNST [7]. This poor outcome clearly underscores the need to get better insight into the exact relationship between TOP2A expression and doxorubicin sensitivity in MPNST and the necessity to reveal new leads for treatment.

A better understanding of the pathobiology of MPNST may lead to the identification of novel treatment targets. Recently, Patel *et al*. reported the upregulation of *Brd4* mRNA and protein levels in a newly developed murine MPNST model [11, 12] based on transplantation of *Nf1*^-/-^,*P53*^-/-^ skin-derived precursor cells into nerves of athymic nude mice [13]. Further investigations inferred a critical role for Brd4 in MPNST pathogenesis as inhibition by shRNAs or by JQ1, a small molecule BET (bromodomain and extraterminal domain) inhibitor, severely impaired *in vitro* growth and *in vivo* tumorigenesis [13]. It was demonstrated that inhibition of Brd4 induced expression of the pro-apoptotic molecule Bim leading to apoptosis in MPNST cells. The BET subfamily of bromodomain proteins to which BRD4 belongs has a role in regulating transcription by RNA polymerase II. The best studied member BRD4 recruits transcriptional regulatory complexes to acetylated chromatin and modulates transcriptional elongation of essential genes involved in cell cycle and apoptosis [14]. In addition, also enhancer of zeste homolog 2 (EZH2) was found upregulated in MPNST compared to neurofibroma and normal nerves [15]. EZH2 is a core element of the polycomb repressive complex 2 (PRC2) a well-known epigenetic modulator of gene expression [16] and is frequently found overexpressed in malignancies or mutated in lymphomas [17]. EZH2 involvement in MPNST pathogenesis was demonstrated by the transient *EZH2* knockdown using si/shRNA or EZH2 inhibition by 3-deazaneplanocin A causing cell cycle arrest and apoptosis in MPNST cells [15, 18]. Evidence is provided for the existence of a novel signaling pathway in MPNST that mediates the effects of EZH2 via miR-30a/30d to karoypherin (importin) beta 1 (KPNB1) [15, 18]. Both EZH2 and BRD4 can be targeted by selective and potent small molecule inhibitors [19, 20] that are currently being evaluated in clinical trials making them appealing targets for the treatment of MPNST.

To further investigate the potential role as treatment targets of the above-mentioned proteins, we investigated the expression level of the target genes in FFPE and fresh frozen sample sets of plexiform neurofibromas and MPNSTs as well as neurofibroma and MPNST cell lines in order to validate the obtained results from the previous studies.

## Materials and methods

### Patients and samples

From the Erasmus MC patient files, nine neurofibroma type 1 patients were selected of which resected plexiform neurofibroma material was present and who developed MPNST. Archival formalin-fixed paraffin-embedded (FFPE) tumor samples of both plexiform neurofibroma and MPNST from the same patient (paired samples) were recovered from the Erasmus MC tissue bank. Fresh frozen samples from plexiform neurofibroma (n = 11), atypical neurofibroma (n = 4) and MPNST (n = 7) were also obtained from the Erasmus MC tissue bank. The FFPE and fresh frozen sample sets do not overlap and were derived from distinct patients. All patients and tumor characteristics are listed in Table 1. For the histopathological diagnosis of MPNST, atypical neurofibroma and plexiform neurofibroma criteria were used as described before [21, 22] in accordance with the 2016 WHO classification of Tumours of the Central Nervous System [23].

**Table 1:** Patient and tumor characteristics.

**Paired FFPE tumor samples (n = 9 pairs)**	
**Gender**	
• Male	6 (66.7%)
• Female	3 (33.3%)
**Age at biopsy/resection NF (years)**	
• Median (range)	28 (5–63)
**Age at biopsy/resection MPNST (years)**	
• Median (range)	27 (14–70)
**Plexiform neurofibroma**	
• Head and neck	1 (11.1%)
• Extremities	3 (33.3%)
• Trunk	5 (55.6%)
**MPNST**	
• Head and neck	1 (11.1%)
• Extremities	4 (44.4%)
• Trunk	4 (44.4%)
**Fresh frozen tumor samples**	
**Plexiform neurofibromas (n = 7)**	
**Gender**	
• Male	4 (57.1%)
• Female	3 (42.9%)
**Age at biopsy/resection (years)**	
• Median (range)	29 (10–63)
**Location**	
• Head and neck	1 (14.3%)
• Extremities	4 (57.1%)
• Trunk	2 (28.6%)
**Atypical neurofibromas (n = 4)**	
**Gender**	
• Male	2 (50%)
• Female	2 (50%)
**Age at biopsy/resection (years)**	
• Median (range)	25.5 (15–43)
**Location**	
• Head and neck	-
• Extremities	4 (100%)
• Trunk	-
**MPNST (n = 11)**	
**Gender**	
• Male	5 (45.5%)
• Female	6 (54.5%)
**Age at biopsy/resection (years)**	
• Median (range)	36 (12–76)
**Location**	
• Head and neck	3 (27.3%)
• Extremities	3 (27.3%)
• Trunk	5 (45.4%)

MPNST; malignant peripheral nerve sheath tumor. NF; plexiform neurofibroma

In short, for the diagnosis of MPNST we used morphological criteria (presence of spindle cells with indistinct cytoplasmic margins and wavy or S-shaped nuclei, arranged in fascicles with alternating cellular and myxoid areas). Immunostaining for S100 was used for identification of a Schwann cell component in the tumors. Atypical neurofibroma was defined by the presence of mitotic figures, and/or cytological atypia, and/or increased cellularity. The combination of all three features, however, defined low grade MPNST. Plexiform neurofibroma involved multiple nerve fascicles and lacked the above mentioned atypical features. Prior to our research the Daily Board of the Medical Ethics Committee Erasmus MC of Rotterdam, The Netherlands, reviewed the research proposal. As a result of this review, the Committee decided that the rules laid down in the Medical Research Involving Human Subjects Act do not apply to this research (MEC-2016-213).

### Cell culture

Human MPNST cell lines ST88-14, 90-8TL, T265 (NF1-associated MPNST) and STS26T (sporadic MPNST) were kindly provided by Dr. Eduard Serra (Institute of Predictive and Personalized Medicine of Cancer/IMPPC, Barcelona, Spain). sNF96.2 and HS53.T were obtained from the ATCC and derived from an NF1-associated MPNST and a cutaneous, NF1-derived, neurofibroma, respectively. Human embryonic kidney (HEK) 293T cells were a kind gift from the department of Genetics, Erasmus MC, Rotterdam, the Netherlands). All cell lines were cultured in DMEM (Gibco Life Technologies) supplemented with 10% fetal bovine serum, 100 IU/ml penicillin and 100 μg/ml streptomycin at 37°C in a humidified 5% CO_2_ atmosphere. All cell lines were regulary monitored for mycoplasma infection and were subjected to authentication by performing a short tandem repeat (STR) DNA analyses and matched, when available, with STR databases. The absence of SUZ12 protein expression in ST88-14 and 90-8TL as reported by de Raedt *et al*. [24] was confirmed by Western blotting (S1 Fig). Similarly, the presence or absence of detectable NF1 protein in the various cell lines was examined (S2 Fig).

### RNA isolation

Total RNA was isolated from cell line pellets and fresh frozen tissues using RNAbee (Tel test Inc., Friendswood, Texas, USA) according to the manufacturer’s instructions. RNA from FFPE tumor samples (5–6 20 μm sections) was isolated using the RecoverAll^TM^ total nucleic acid isolation kit (Ambion/Life Technologies). RNA quality and quantity were checked using a Nanodrop-1000 (Nanodrop Technologies).

### Quantitative RT-PCR

cDNA was synthesized from 250 ng of total RNA using TaqMan^®^ Reverse Transcription Reagents (ThermoFisher Scientific). The mRNA expression levels of target genes and housekeepers were determined by real time PCR using TaqMan^®^ Universal PCR Master Mix and specific Assay-On-Demand products (ThermoFisher Scientific/Applied Biosystems) using an ABI 7500 Real-Time PCR machine. The following assays were used *EZH2* (Hs01016789_m1), *TOP2A*(Hs01032137_m1), *BRD4*(Hs04188087_m1). Expression of *EZH2*, *TOP2A* and *BRD4* were normalized using *PPIA* (Pedersen *et al*, 2014) (Hs99999904_m1) using the comparative C_T_ method [25]. Each tumor or cell line RNA sample was measured in duplicate after which the data were analyzed using SDS software (Applied Biosystems). Statistical significance (p<0.05) was determined on the normalized expression values of the paired FFPE samples using a paired Student t-test.

### Protein lysates, SDS-PAGE and Western blotting

Total protein was extracted from cells using lysis buffer (50 mM Tris-HCl pH 7.5, 50 mM NaCl, 10% glycerol, 1% NP-40, 0.5% Na-deoxycholate, 1 mM Na_3_VO_4_, 20 mM NaF, 1 mM Pefabloc) supplemented with a cocktail of protease inhibitors. Protein concentration was quantified using a Bradford assay (Bio-Rad, Veenendaal, The Netherlands). Equal amounts of total protein (15–20 μg/lane) were subjected to SDS-PAGE and subsequently transferred to a PVDF membrane by electroblotting. Remaining protein binding sites of the membrane were blocked in PBS, 0.05% Tween 20 (PBS/Tween) containing 5% non-fat dried milk. Primary antibody incubations were carried out in the same buffer with the following primary antibodies: mouse monoclonal anti-EZH2 (1:1000, NCL-L-EZH2, Leica Microsystems;), rabbit monoclonal anti-SUZ12 (1:1000, D39F6, Cell Signaling Technology), rabbit polyclonal anti-BRD4 (1:10000, A301-985A100, Bethyl Laboratories, Inc), rabbit monoclonal anti-TOP2A (1:1000, D10G9, Cell Signaling Technology), rabbit monoclonal anti-NF1 (1:1000, D7R7D, Cell Signaling Technology), rat monoclonal anti-tubulin (1: 4000, YL1/2, Abcam) and mouse monoclonal anti-β-actin (1:10000, A5441, Sigma-Aldrich). HRP conjugated goat-anti-rabbit, goat-anti-mouse and goat-anti-rat were used as secondary antibodies. Enhanced chemiluminiscence (SuperSignal West Pico Chemiluminescent Substrate, Thermo Scientific, Rockford, IL, USA) was used to visualise the signal in a ChemiDoc MP Imager (Bio-Rad, Veenendaal, The Netherlands). Protein expression was quantitated using ImageJ, a public domain Java-based image processing program [26]. Each Western blot was replicated at least three times, depicted are representative blots.

### In vitro cytotoxicity assay

*In vitro* cytotoxicity of the BET inhibitor JQ1 (BioVision Inc, Milpitas, CA, USA), and the anthracycline doxorubicin (Pharmachemie, Haarlem, The Netherlands) were determined by a sulforhodamine B (SRB) assay essentially as described by Keepers *et al*. [27]. In brief, on day 0 cells were plated in 96-well flat bottom microtiter plates. On day 1 a ten-step, two-fold dilution series was prepared and added to the cells resulting in a highest concentration of 2500 nM for JQ1 and 500 ng/ml for doxorubicin. Every dilution was assayed in quadruplicate. After 48–72 hours the assay was terminated, the cells fixed with 10% trichloroacetic acid in PBS for 1 h at 4°C. After at least four washes with tap water the cells remaining in the wells were stained with 0.4% SRB in 1% acetic acid for at least 15 min at RT. Subsequently the unbound stain was removed by 4 washes in 1% acetic acid. Plates were air-dried and bound stain was dissolved in 150 μl of 10 mM Tris-base. Staining was quantified by measuring the absorbance at 540 nm in a spectrophotometer. Concentration-response curves were generated and IC_50_ values were calculated by the use of Deltasoft 3 software.

### *EZH2* siRNA mediated knockdown

Twentyfour hours prior to transfection the 90-8TL and T265 cell lines were plated in a 24-well plate in duplicate at such a concentration that the next day the wells reach 70–80% confluency. Cells were transfected with either a *EZH2*-specific siRNA (Qiagen, FlexiTube siRNA SI02665166) or a negative control scrambled siRNA (Qiagen, SI03650325) at a concentration of 50 nM using the DharmaFECT1 transfection reagent (Dharmacon/Thermo Scientific) as recommended by the manufacturer. Twentyfour hours post-transfection the medium was replaced with standard culture medium and cell density as a measure for proliferation was assessed by SRB staining at 24, 48 and 72 hours after transfection.

## Results

### Human *BRD4* mRNA levels are not increased in MPNST compared to neurofibromas

**Fig 1:**
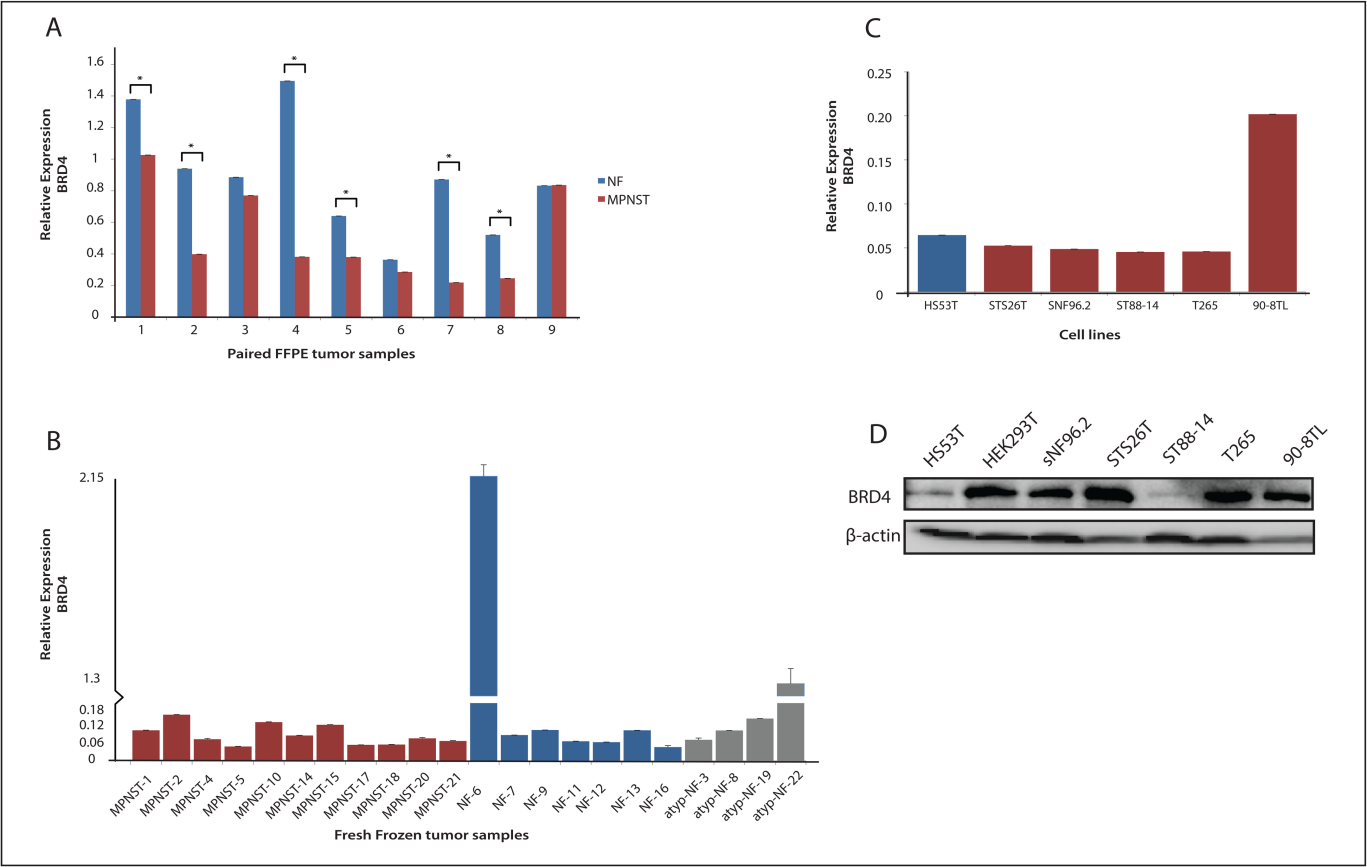
Expression level of *BRD4* in human neurofibroma and MPNST samples and cell lines. (**A**) qRT-PCR was used to determine mRNA levels of *BRD4* in paired plexiform neurofibroma (NF, blue, n = 9) and MPNST (red, n = 9) formalin-fixed paraffin-embedded tumor samples, each pair being derived from the same NF1 patient. Asterisk indicates P<0.05. (**B**) qRT-PCR was used to determine mRNA levels of *BRD4* in fresh frozen MPNST (red, n = 11), plexiform neurofibroma (blue, n = 7) and atypical neurofibroma (grey, n = 4). (**C**) qRT-PCR was used to determine mRNA levels of *BRD4* in a cell line panel: Hs53.T neurofibroma cell line (blue) and STS26T, sNF96.2, ST88-14, T265 and 90-8TL MPNST cell lines (red) (**D**) Western blot displaying BRD4 protein expression in cell line panel and HEK293T. ẞ-actin levels are shown as a loading control.

**Fig 2:**
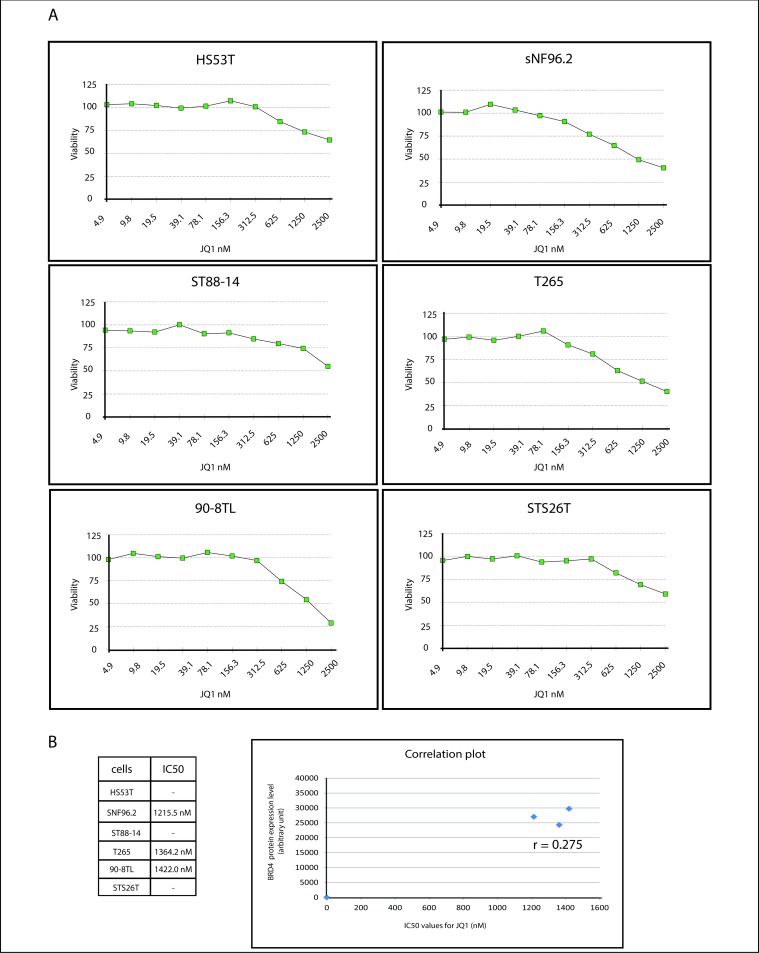
Sensitivity of neurofibroma and MPNST cell lines to the BET bromodomain inhibitor JQ1. (**A**) An *in vitro* cytotoxicity assay (SRB assay) was used to determine IC_50_ values (nM) for the BET bromodomain inhibitor JQ1 of neurofibroma and MPNST cell lines after a 72h exposure to the drug. Graphs show cell viability as a function of JQ1 concentration. Depicted is the average viability (n = 4) of a representative experiment. (**B**) Listing of calculated IC_50_ values and correlation plot, with BRD4 protein expression levels on the Y-axis and IC_50_ values for JQ1 on the X-axis. Pearson correlation coefficient is depicted in the graph.

In the search for targetable alterations in MPNST Patel *et al*. reported a potential pathogenic role of a BET bromodomain family member (*Brd4*) in an MPNST mouse model. Inhibition of Brd4, which was found highly upregulated in MPNST, induced increased expression of the pro-apoptotic molecule Bim inducing apoptosis in MPNST cells and tumor shrinkage [13]. We examined *BRD4* mRNA expression by qRT-PCR in a series of nine paired human MPNST and plexiform neurofibroma FFPE samples, each pair derived from the same patient (Fig 1A). To rule out that degradation of the total RNA isolated from the archival samples impairs accurate quantitation we also determined *BRD4* mRNA levels in a set of fresh frozen plexiform neurofibromas (n = 7), atypical neurofibromas (n = 4) and MPNST (n = 11) (Fig 1B). Both in the neurofibroma-MPNST pairs as well as in the fresh frozen samples we did not detect *BRD4* overexpression in the MPNST samples (Fig 1A and 1B). The paired sample analyses indicated significantly higher *BRD4* mRNA levels in 6 of the neurofibromas compared to their corresponding MPNST whereas in most fresh frozen samples there was no significant difference in *BRD4* mRNA levels between (atypical) plexiform neurofibromas and MPNSTs. It must be noted, however, that mRNA levels may not be indicative for protein levels as in most MPNST cell lines *BRD4* mRNA levels were similar but BRD4 protein levels varied considerable (cf. Fig 1C and 1D). To further investigate whether BRD4 can serve as a target for treatment we determined the sensitivity of our cell line panel consisting of a neurofibroma and 5 MPNST cell lines, to the BET bromodomain inhibitor JQ1 (Fig 2A). In an *in vitro* cytotoxicity assay the cell lines were exposed to increasing concentrations of JQ1 for 72 hours. Most MPNST cell lines did not display a clearly increased sensitivity to JQ1 compared to the neurofibroma cell line. The MPNST cell lines sNF96.2, T265 and 90-8TL expressed approximately equal levels of BRD4 protein and displayed similar sensitivity to JQ1 (Fig 2B). ST88-14 another NF1-derived MPNST cell line expressed relatively low BRD4 protein levels and was accordingly found less sensitive to JQ1. In contrast the sporadic MPNST cell line STS26T harbors high levels of BRD4 protein but is relatively insensitive to JQ1. For these cell lines we were not able to calculate IC_50_ values (Fig 2B).

### EZH2 levels are increased in MPNST compared to neurofibromas but do not affect cellular proliferation

**Fig 3:**
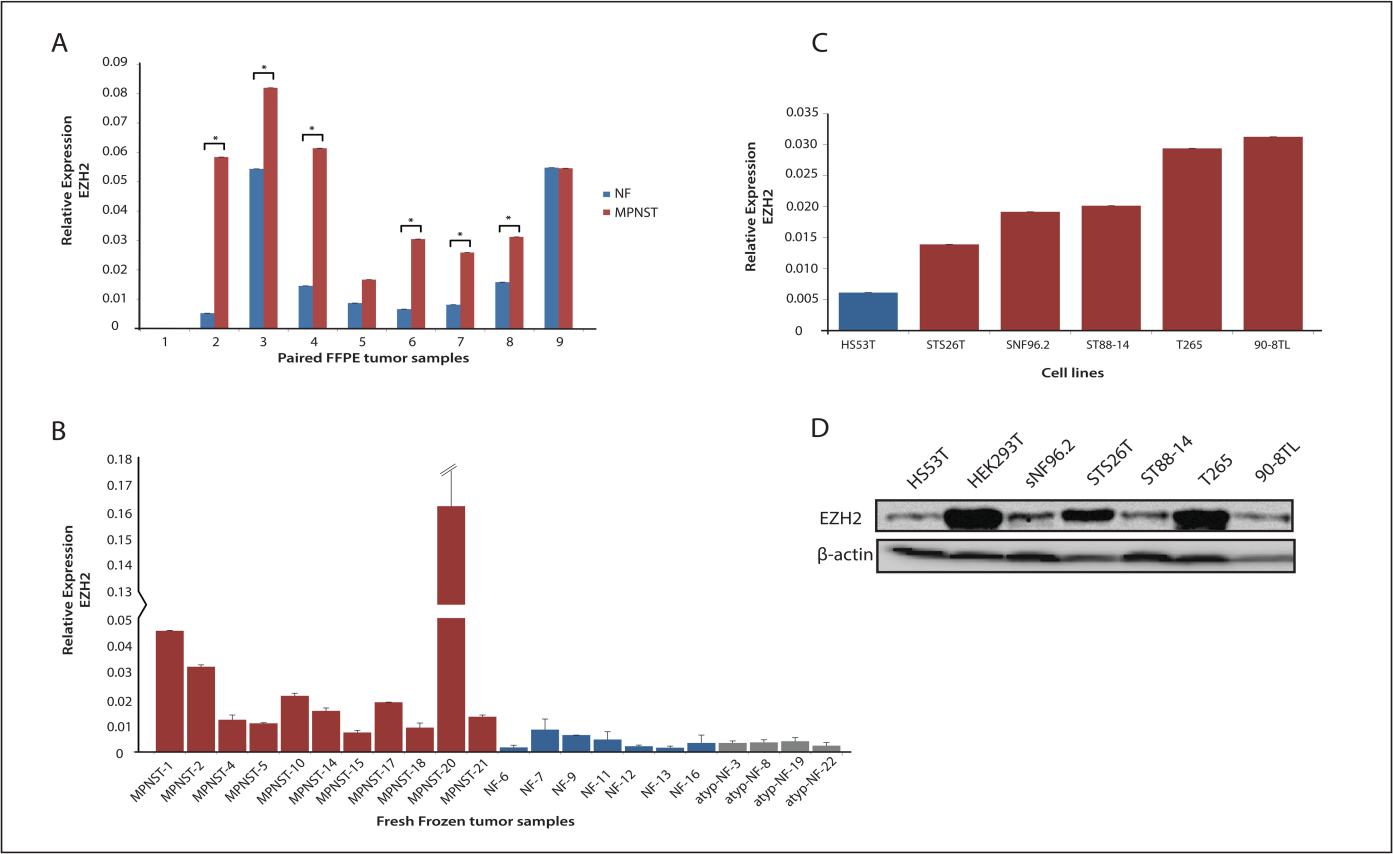
Expression level of *EZH2* in human neurofibroma and MPNST samples and cell lines. (**A**) qRT-PCR was used to determine mRNA levels of *EZH2* in paired plexiform neurofibroma (NF, blue, n = 9) and MPNST (red, n = 9) formalin-fixed paraffin-embedded tumor samples, each pair being derived from the same NF1 patient. Asterisk indicates P<0.05. (**B**) qRT-PCR was used to determine mRNA levels of *EZH2* in fresh frozen MPNST (red, n = 11), plexiform neurofibroma (blue, n = 7) and atypical neurofibroma (grey, n = 4). (**C**) qRT-PCR was used to determine mRNA levels of *EZH2* in a cell line panel: Hs53.T neurofibroma cell line (blue) and STS26T, sNF96.2, ST88-14, T265 and 90-8TL MPNST cell lines (red). (**D**) Western blot displaying EZH2 protein expression in cell line panel and HEK293T. ẞ-actin levels are shown as a loading control.

**Fig 4:**
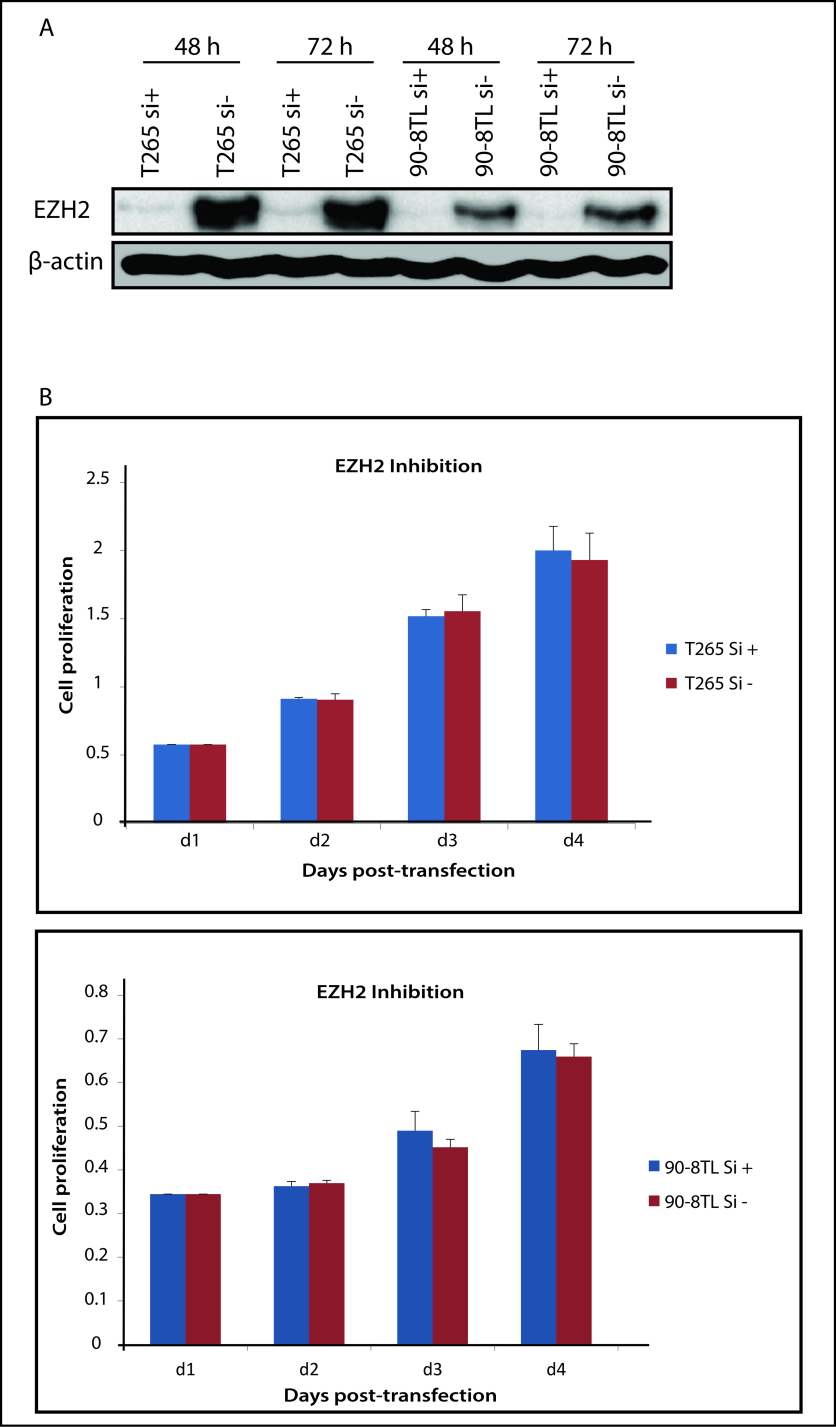
siRNA mediated knockdown of EZH2 and its effect on cell proliferation. (**A**) Western blot showing the effect of EZH2 siRNA (si+) or a scrambled control siRNA (si-) on EZH2 protein levels in T265 and 90-8TL at 48h and 72 h post-transfection. (**B**) Cell proliferation monitored in time after transfection of T265 and 90-8TL with *EZH2* siRNA (si+) or a scrambled control siRNA (si-). ẞ-actin levels are shown as a loading control.

Nuclear EZH2 levels were reported to be induced in MPNST compared to neurofibromas and normal nerves as measured by immunohistochemistry [15]. Our observations support these results as the *EZH2* mRNA levels were significantly increased in the MPNST samples from 6 out of 9 plexiform neurofibroma/MPNST pairs (Fig 3A). Also in RNA isolated from fresh frozen neurofibroma and MPNST samples *EZH2* mRNA levels appeared on average to be 8-fold higher in MPNST than in (atypical) plexiform neurofibromas (Fig 3B). Similarly, all the MPNST cell lines displayed relatively high *EZH2* mRNA levels compared to the neurofibroma cell line (Fig 3C). At a protein level, as judged by Western blot, EZH2 also seems more highly expressed in the MPNST cell lines although it is clear that protein expression and mRNA levels do not always perfectly match (Fig 3D). Next, we investigated whether EZH2 inhibition exerts an anti-proliferation activity as was previously reported [15]. Both T265 and 90-8TL MPNST cells were transiently transfected with an *EZH2* siRNA and a scrambled siRNA control for comparison. EZH2 protein levels were significantly reduced by the *EZH2* siRNA treatment at 48–72 h after transfection (Fig 4A). However, despite the clearly decreased EZH2 levels no significant inhibition of cell proliferation was observed (Fig 4B).

### Relative high expression of TOP2A in MPNST is associated with doxorubicin sensitivity

**Fig 5:**
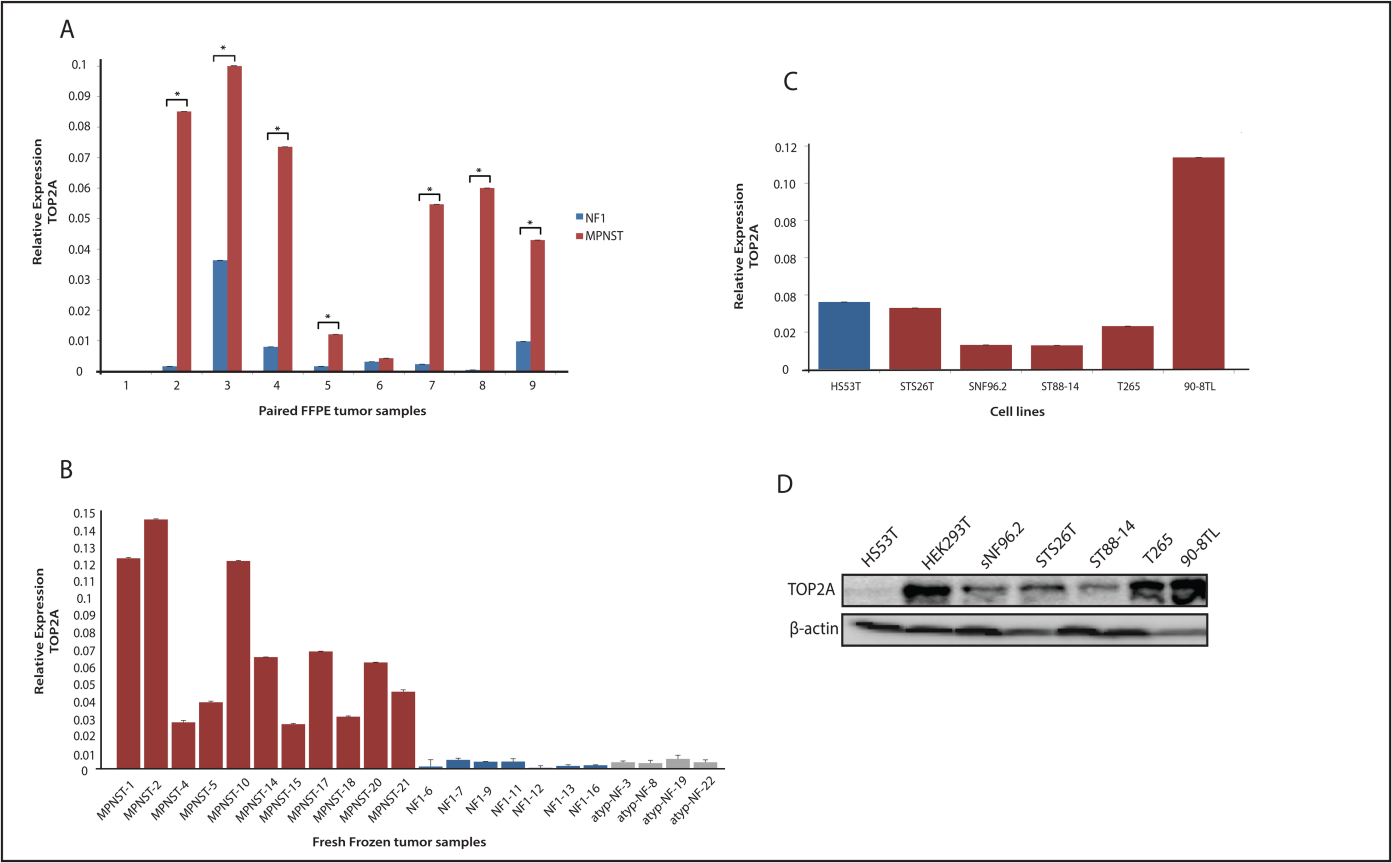
Expression level of TOP2A in human neurofibroma and MPNST samples and cell lines. (**A**) qRT-PCR was used to determine mRNA levels of *TOP2A* in paired plexiform neurofibroma (NF, blue, n = 9) and MPNST (red, n = 9) formalin-fixed paraffin-embedded tumor samples, each pair being derived from the same NF1 patient. Asterisk indicates P<0.05. (**B**) qRT-PCR was used to determine mRNA levels of *TOP2A* in fresh frozen MPNST (red, n = 11), plexiform neurofibroma (blue, n = 7) and atypical neurofibroma (grey, n = 4). (**C**) qRT-PCR was used to determine mRNA levels of *TOP2A* in a cell line panel: Hs53.T neurofibroma cell line (blue) and STS26T, sNF96.2, ST88-14, T265 and 90-8TL MPNST cell lines (red). (**D**) Western blot displaying TOP2A protein expression in cell line panel and HEK293T. ẞ-actin levels are shown as a loading control.

**Fig 6:**
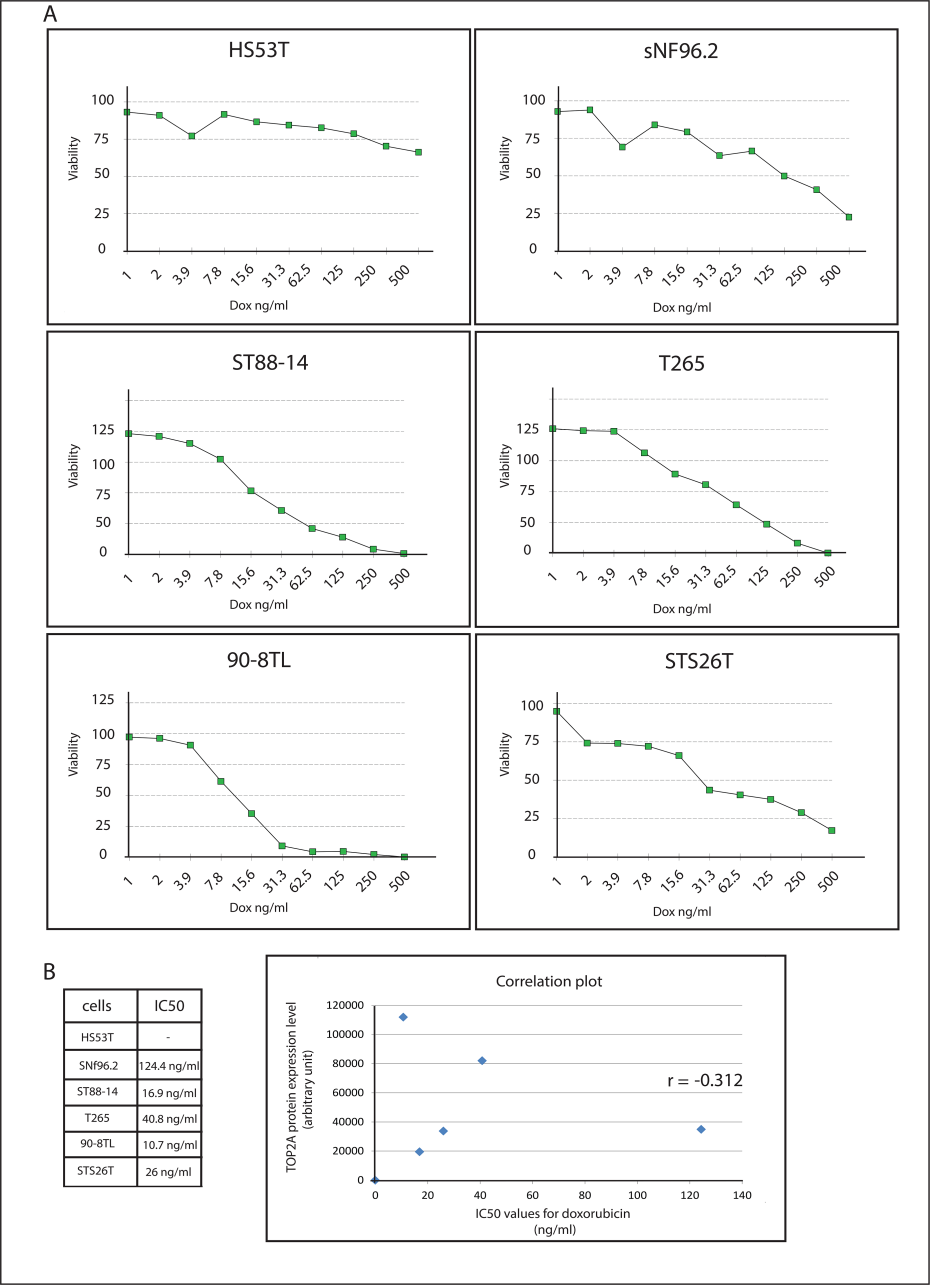
Sensitivity of neurofibroma and MPNST cell lines to doxorubucin. (**A**) An *in vitro* cytotoxicity assay (SRB assay) was used to determine IC_50_ values (ng/ml) for doxorubucin of neurofibroma and MPNST cell lines after a 48h exposure to the drug. Graphs show cell viability as a function of doxorubucin concentration. Depicted is the average viability (n = 4) of a representative experiment. (**B**) Listing of calculated IC_50_ values and correlation plot, with TOP2A protein expression levels on the Y-axis and IC_50_ values for doxorubicin on the X-axis. Pearson correlation coefficient is depicted in the graph.

To verify whether TOP2A expression levels are increased in MPNST as was reported in the literature [9, 10] we determined the *TOP2A* mRNA levels in our paired FFPE and fresh frozen plexiform neurofibroma/MPNST sample sets. In both panels *TOP2A* mRNA expression was clearly induced in MPNST when compared to the levels detected in plexiform neurofibromas. In 7 out 9 paired FFPE samples *TOP2A* levels were significantly increased in the MPNST samples (Fig 5A). In the fresh frozen sample set *TOP2A* mRNA levels were on average 24-fold higher in the MPNST than in the plexiform neurofibromas (Fig 5B). In the cell line panel *TOP2A* mRNA levels in the MPNST cell lines were mostly equal or lower than the levels measured in the neurofibroma cell line Hs53.T, only the MPNST 90-8TL cell line exhibited relatively high *TOP2A* levels (Fig 5C). At the protein level, however, all MPNST cell lines displayed markedly higher TOP2A expression than the Hs53.T cells (Fig 5D). To examine whether the relatively high MPNST TOP2A levels translate into sensitivity to the TOP2A targeting chemotherapeutic drug doxorubicin we determined the sensitivity of the cell lines to this drug using an *in vitro* cytotoxicity (SRB) assay. All four NF1-associated MPNST cell line (sNF96.2, ST88-14, T265 and 90-8TL) and one sporadic MPNST cell line (STS26T) were more sensitive to doxorubicin than the neurofibroma Hs53.T cells, many of them displaying IC_50_ values of less than 50 ng/ml (Fig 6A and 6B). A comparison of TOP2A protein expression levels and the calculated IC_50_ values of the cell lines indicated a correlation, although not very strong, of TOP2A levels and doxorubicin sensitivity (Fig 6B).

## Discussion

Given the limited number of therapeutic options for advanced MPNST patients, the identification of novel drug targets and the development of new treatments and treatment strategies is urgently needed. In this study we analyzed the expression level of three potential drug targets BRD4, EZH2, and TOP2A in selected human MPNST and neurofibroma samples from the Erasmus MC tissue bank. Our sample set included both fresh frozen samples and a set of nine paired FFPE samples consisting of plexiform neurofibromas and MPNST that were resected from the same patient.

With respect to BRD4, it has been shown that inhibition of this protein profoundly suppresses MPNST tumorigenesis and tumor cell growth in a murine MPNST model [13]. To confirm this putative key role of BRD4 in human MPNST pathogenesis, we evaluated the expression level of *BRD4* in plexiform neurofibromas and MPNST samples. In addition, we studied the effect of BRD4 modulation by JQ1 on the cell viability of MPNST cell lines. In contrast to what has been reported for the MPNST mouse model [13], we did not find evidence for an increased expression of *BRD4* in human MPNST samples when compared to plexiform neurofibromas. It must be noted, however, that we only examined a limited set tumor samples due to the rarity of MPNST. Additionally, in order to deal with tumor heterogeneity, it may be useful to examine multiple biopsies from the same tumor. Nevertheless our analyses of *BRD4* expression, either of FFPE or fresh frozen samples, do not indicate an overexpression in MPNST. In contrast, previously reported overexpression of *EZH2* and *TOP2A* in MPNST could be convincingly demonstrated in our sample sets, using similar RT-PCR assays, indicating RNA quality is good. Alternatively, our inability to confirm *BRD4* overexpression in the human MPNST setting may indicate that data acquired with genetically engineered animal models cannot always be easily translated to the human situation. It might be that these models do not recapitulate the full complexity of human cancers and/or there are unrecognized fundamental cross-species differences in the process of tumorigenesis [28, 29]. Moreover, BRD4 inhibition by JQ1 treatment in our panel of MPNST cell lines indicated that they were less sensitive to JQ1 than the primary murine skin-derived precursors (*Nf1*^*-/-*^, *P53*^*-/-*^) and MPNST cells derived thereof which display IC_50_ values of < 400 nM [13]. Although Patel *et al*. did use the human S462 MPNST cell line they did not present a dose-response curve from which an IC_50_ value could be deduced making a direct comparison with our results difficult. Likewise Patel *et al*. did not validate their findings regarding Brd4 overexpression in clinical tumor samples. Interestingly, de Raedt and colleagues provided evidence that BRD4 inhibition by JQ1 exerted only a modest, cytostatic effect on human MPNST cell lines and that only the combination of JQ1 with PD-901, a MEK-inhibitor, caused a tumor growth inhibition and regression [24].

Zhang *et al*. demonstrated that EZH2 is overexpressed in MPNST and fulfils a key role in tumorigenesis [15, 18]. Both downregulation of *EZH2* by si/shRNA or pharmacological inhibition of EZH2 in the S462 (NF1-derived MPNST) and MPNST724 (spontaneous MPNST) cell lines severely affected cellular proliferation rates, induced apoptosis and interfered with tumor formation in an MPNST724 xenograft model. We do confirm that *EZH2*, at least at the mRNA level, is more abundantly expressed in MPNST than in plexiform neurofibromas. However, when we examined the consequences of EZH2 downregulation on cellular proliferation in 90-8TL and T265 we did not observe any inhibitory effect, despite a significant EZH2 knockdown. It might be that the cell lines used by Zhang *et al*. respond differently to EZH2 knockdown or inhibition than the NF1-derived MPNST cell lines 90-8TL and T265 that we examined. It may be that knockdown of EZH2 is compensated for by other members of the PRC2 complex and/or the related EZH1. Our findings, however, do suggest that EZH2 functions may be dependent on cellular context. Importantly, it was recently reported that a substantial number of MPNST, irrespective of their origin (NF1-derived, spontaneous or radiation induced) exhibit an inactivated PRC2 complex due to somatic loss-of-function mutations in *SUZ12* and *EED* [24, 30, 31]. Both SUZ12 and EED—just as EZH2—are integral parts of the PRC2 complex. It is not yet known what the consequences of such a PRC2 inactivation are for the remaining unaffected PRC2 complex subunits like EZH2. Is EZH2 still present in a protein complex and is EZH2 capable of fulfilling a biological role in this context or on its own? Perhaps the discrepancy between our findings and those of Zhang *et al*. [15] can be explained by different levels of PRC2 complex inactivation in the cell lines used. Translated to the clinic this would imply that before targeting EZH2 in the context of MPNST it is imperative to verify whether the PRC2 complex is in fact inactivated e.g. by determining the absence of H3K27 trimethylation (H3K27me_3_) in the tumor tissue. Only MPNST patients that display an active PRC2 complex may benefit from EZH2 inhibition.

The enzyme TOP2A functions in maintaining DNA topology after replication. The cellular abundance of TOP2A is reported to determine the efficacy of anthracycline based chemotherapy in various cancers [32–37]. The anthracycline doxorubicin, a widely used anticancer agent, can interfere with the catalytic cycle of TOP2A either by preventing its binding to DNA or by trapping TOP2A cleavage complexes and blocking DNA religation generating double strand DNA breaks [8]. *TOP2A* levels in MPNST were reported to be upregulated due to amplification of the *TOP2A* gene [9, 10]. Our results verified the abundant expression of *TOP2A* in MPNST and may explain why doxorubicin is widely used in the treatment of advanced MPNST patients. Though in general outcomes are poor, some patients may derive durable benefit from doxorubicin based treatment [7]. When we determined the sensitivity of our neurofibroma and MPNST cell line panel for doxorubicin we observed that the MPNST cell lines exhibited the highest sensitivity in agreement with their higher TOP2A levels. Still the outcome of doxorubicin treatment in the clinic is poor for most MPNST patients perhaps due to the rapid activation of drug resistance mechanisms that diminish the efficacy of this chemotherapy.

From this study, we tentatively conclude that the potential for effective therapeutic intervention in MPNST by targeting BRD4, EZH2 and TOP2A individually, is limited. However, this does not preclude the use of inhibitors in certain subpopulations of patients and/or in combination therapies. We strongly encourage other research groups to validate our findings and are in favor of clinical studies involving patients as only these will ultimately prove the true value of BRD4, EZH2 and TOP2A inhibitors in the MPNST setting. Last but not least further investigations are needed into the biology of MPNST to identify additional druggable disease drivers for novel therapeutic strategies.

## Supporting information

S1 Fig Neurofibroma and MPNST cell lines. (**A**) Overview of neurofibroma and MPNST cell lines. (**B**) Western blot displaying SUZ12 protein expression in neurofibroma and MPNST cell line panel and HEK293T. ẞ-actin levels are shown as a loading control.
(TIF)

S2 Fig NF1 expression in neurofibroma and MPNST cell lines. Western blot displaying NF1 protein expression in neurofibroma and MPNST cell line panel and the non-small cell lung cancer cell lines NCI-H460 and NCI-H1299. Tubulin levels are shown as a loading control.
(TIF)

S3 Fig BRD4 protein expression in neurofibroma and MPNST cell line panel. Uncropped blot related to Fig 1D.
(TIF)

S4 Fig EZH2 protein expression in neurofibroma and MPNST cell line panel. Uncropped blot related to Fig 3D.
(TIF)

S5 Fig EZH2 protein expression upon siRNA mediated knockdown. Uncropped blot related to Fig 4A.
(TIF)

S6 Fig TOP2A and SUZ12 protein expression in neurofibroma and MPNST cell line panel. Uncropped blot related to Fig 5D (TOP2A) and S1 Fig. (SUZ12).
(TIF)

S1 Table Ct and normalized expression values of individual data points. Excel file containing RT-PCR data related to Fig 1A–1C; Fig 3A–3C and Fig 5A–5C.
(XLSX)
