# Supplementary figures and images for: Expression and inhibition of BRD4, EZH2 and TOP2A in neurofibromas and malignant peripheral nerve sheath tumors
Source: PLoS One. 2017 Aug 15;12(8):e0183155. doi: 10.1371/journal.pone.0183155 (PMC5557548; doi:10.1371/journal.pone.0183155)

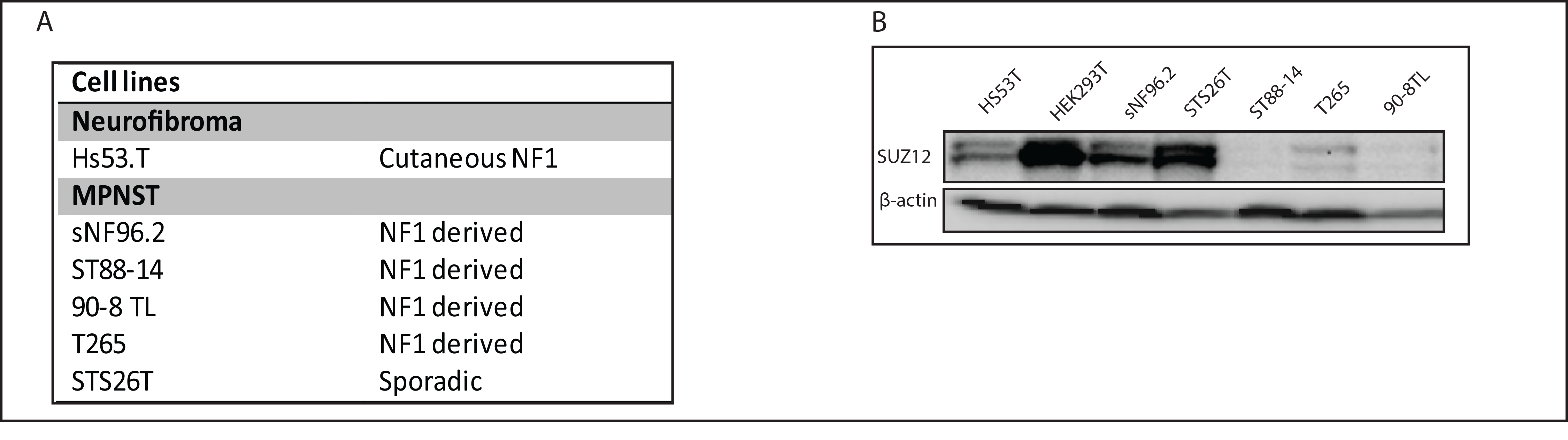

Supplement: S1 Fig — (A) Overview of neurofibroma and MPNST cell lines. (B) Western blot displaying SUZ12 protein expression in neurofibroma and MPNST cell line panel and HEK293T. ẞ-actin levels are shown as a loading control. (TIF) [file pone.0183155.s001.tif]

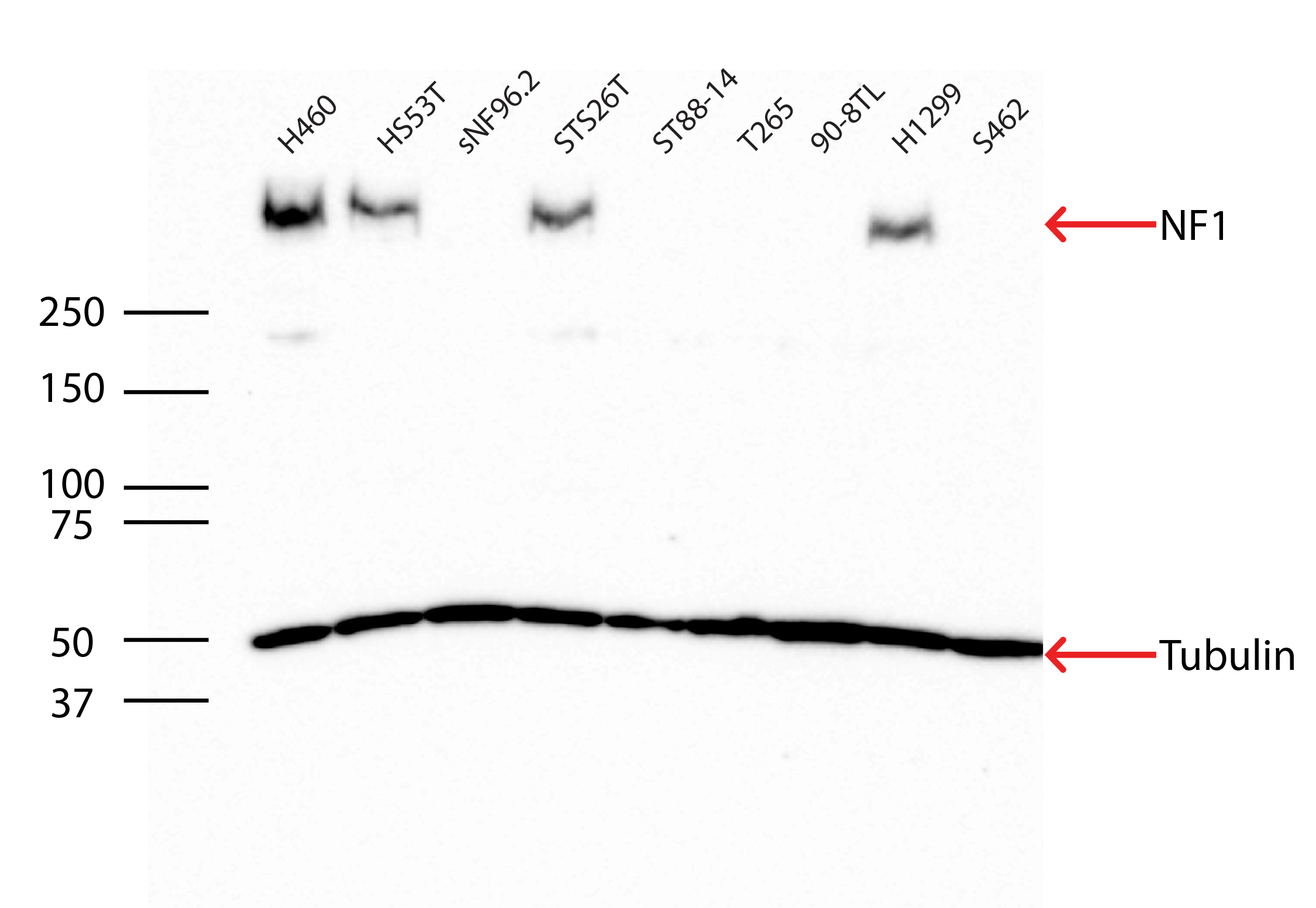

Supplement: S2 Fig — Western blot displaying NF1 protein expression in neurofibroma and MPNST cell line panel and the non-small cell lung cancer cell lines NCI-H460 and NCI-H1299. Tubulin levels are shown as a loading control. (TIF) [file pone.0183155.s002.tif]

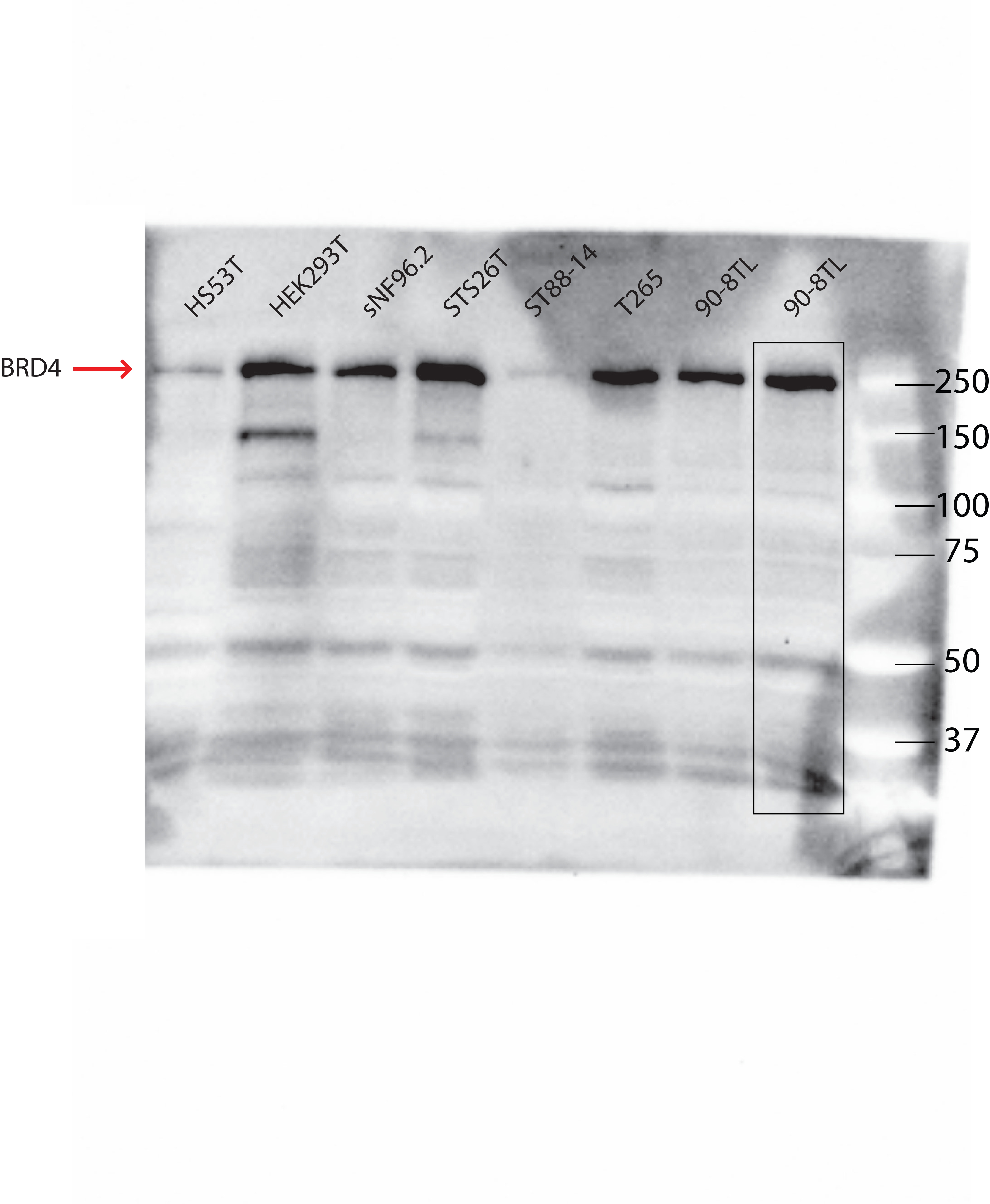

Supplement: S3 Fig — Uncropped blot related to Fig 1D. (TIF) [file pone.0183155.s003.tif]

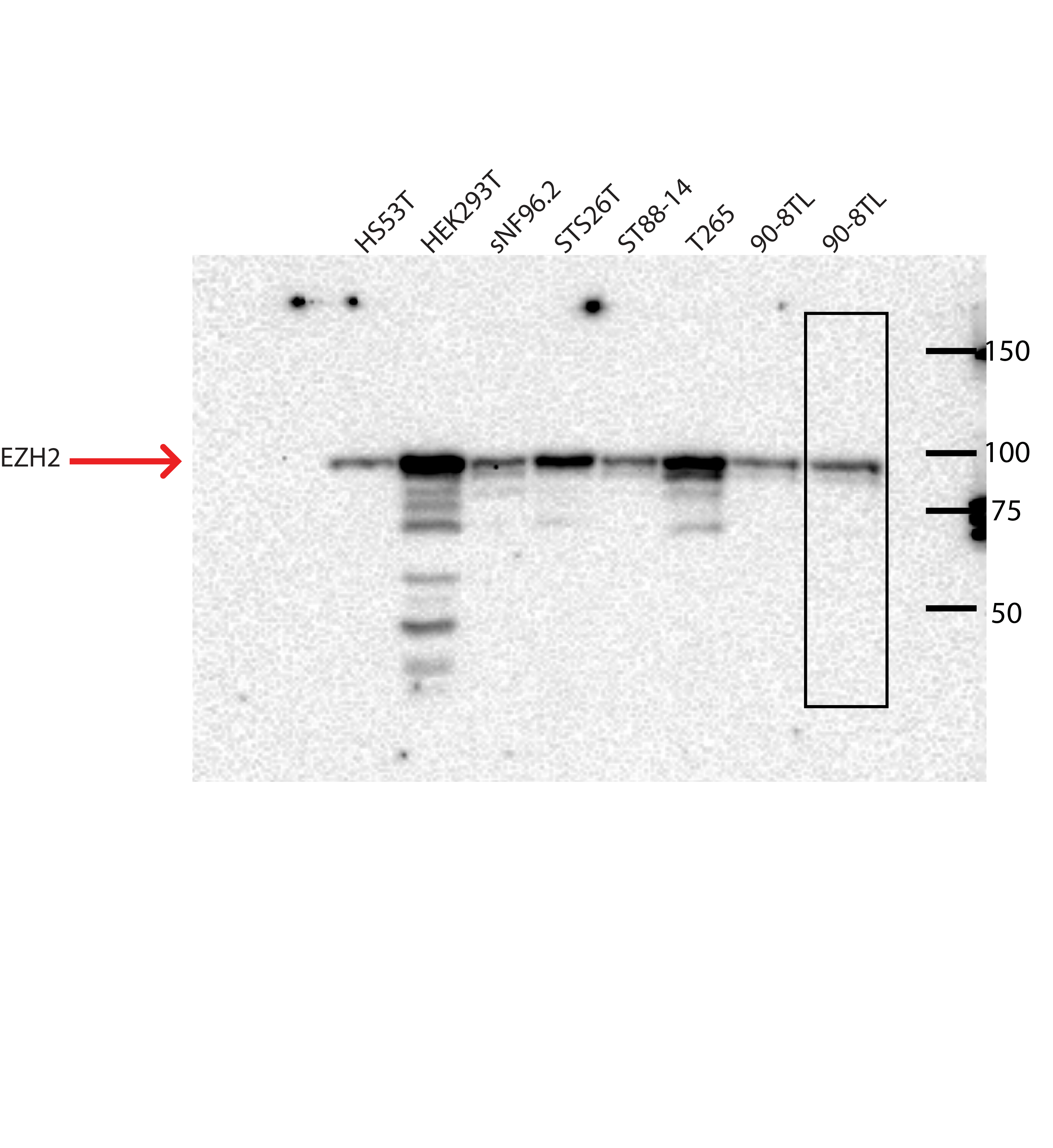

Supplement: S4 Fig — Uncropped blot related to Fig 3D. (TIF) [file pone.0183155.s004.tif]

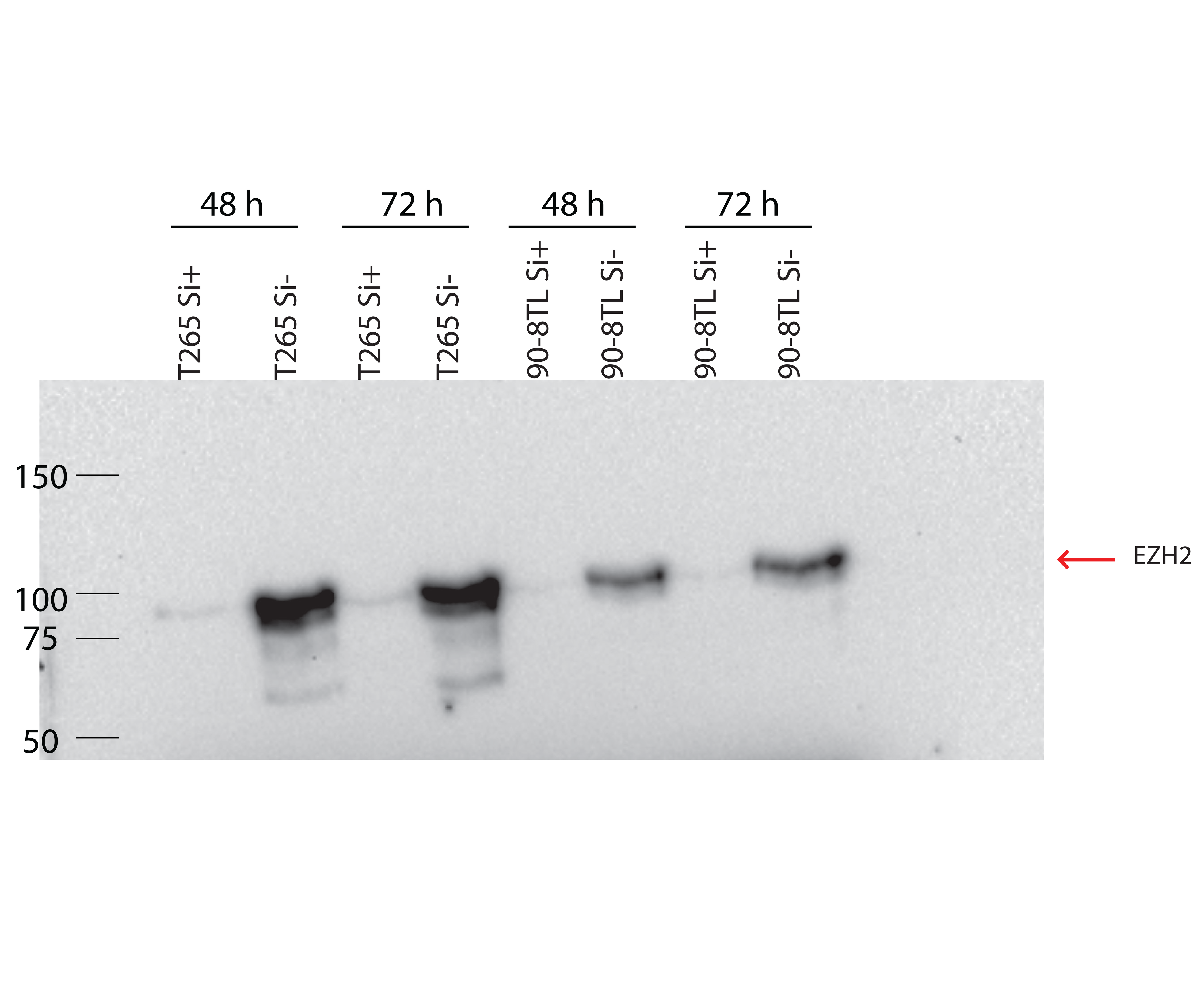

Supplement: S5 Fig — Uncropped blot related to Fig 4A. (TIF) [file pone.0183155.s005.tif]

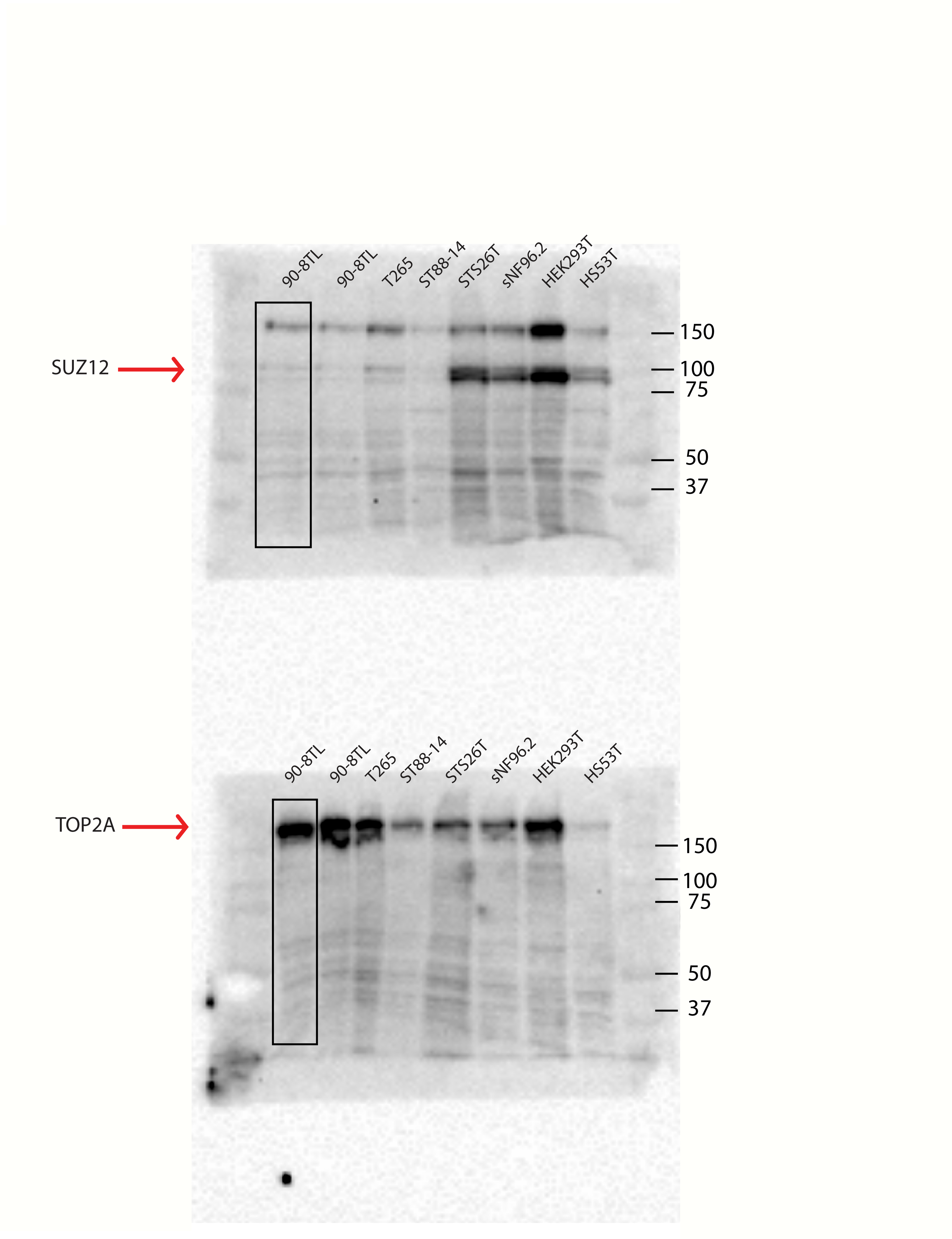

Supplement: S6 Fig — Uncropped blot related to Fig 5D (TOP2A) and S1 Fig. (SUZ12). (TIF) [file pone.0183155.s006.tif]
